# Dynein axonemal intermediate chain 2 plays a role in gametogenesis by activation of Stat3

**DOI:** 10.1111/jcmm.13945

**Published:** 2018-11-01

**Authors:** Zhaojuan Yang, Bo Xu, Xiaopeng Hu, Xiaoying Yao, Yunhui Tang, Cuifeng Qian, Shuzeng Wang, Haifeng Chen, Xiaohui Bai, Ji Wu

**Affiliations:** ^1^ Key Laboratory for the Genetics of Developmental and Neuropsychiatric Disorders (Ministry of Education) Bio‐X Institutes Shanghai Jiao Tong University Shanghai China; ^2^ Obstetrics and Gynecology Hospital Fudan University Shanghai China; ^3^ School of Life Sciences and Biotechnology Shanghai Jiao Tong University Shanghai China; ^4^ Key Laboratory of Fertility Preservation and Maintenance of Ministry of Education Ningxia Medical University Yinchuan China; ^5^ Shanghai Key Laboratory of Reproductive Medicine Shanghai China

**Keywords:** *Dnaic2*, oogenesis, spermatogenesis, Stat3

## Abstract

We previously identified the mouse dynein axonemal intermediate chain 2 (*Dnaic2*) gene. This gene expresses a component of the axonemal dynein complex that functions in cilia or flagella. We found that overexpression of *Dnaic2* results in female subfertility and male infertility. In this study, we generated *Dnaic2* knockdown (KD) mice and identified the potential regulatory mechanisms involved in *Dnaic2* function. For phenotype analysis, we found that body weight was lighter and size was smaller in *Dnaic2* KD mice than in wild‐type mice. A total of 45% of these *Dnaic2* KD mice were infertile due to sperm abnormalities in males, or had oocyte abnormalities and pathological changes in the tunica mucosa in the oviduct of females. Moreover, *Dnaic2* overexpression enhanced the expression of proliferating cell nuclear antigen (PCNA) in the ovaries, which suggested that *Dnaic2* stimulated proliferation of cells in the ovaries. However, PCNA expression in the testis of *Dnaic2*‐overexpressed mice was lower than that in controls. Additionally, the ratio of *Bax*/B‐cell lymphoma‐2*(Bcl‐2)* in the testis of *Dnaic2*‐overexpressed mice was higher than that in controls, which suggested that *Dnaic2* inhibited cellular proliferation in the testis. To examine the molecular action of *Dnaic2*, immunoprecipitation analysis was used and showed that Dnaic2 protein interacted with signal transducer and activator of transcription 3 (Stat3). Molecular modelling analysis showed that Dnaic2 bound with the linker and SH2 domains of Stat3. Furthermore, overexpression of *Dnaic2* promoted phosphorylation of Stat3. In conclusion, our study suggests that *Dnaic2* plays a role in oogenesis and spermatogenesis by activation of Stat3.

## 1 INTRODUCTION

Gametogenesis, including oogenesis and spermatogenesis, are critical processes in the development of mammalian reproduction. Any defects that occur in these processes result in infertility of adult animals. In mammalian ovaries, oogenesis is closely associated with folliculogenesis, which is tightly regulated by cell survival and death signals that are partly derived from oocytes.^1^, ^2^ Follicular atresia and degeneration of oocytes are the fate of most follicles and oocytes. During spermatogenesis, spermatogonial cells develop into spermatids through maturation of proliferation and differentiation. Approximately 75% of these developed germ cells are generally lost by apoptosis or degeneration.^3^ Moreover, regulation of proliferation and cell death of type A spermatogonia determines the number of differentiated spermatogonia that can enter spermatogenesis.^4^ Therefore, cellular proliferation and apoptosis in gametogenesis are essential issues for mammalian reproductive development. Most signals or proteins that are involved in cellular proliferation and apoptosis, such as Bax, B‐cell lymphoma‐2 (Bcl‐2), and stem cell factor (SCF)/c‐kit, play important roles in gametogenesis.^5^, ^6^, ^7^, ^8^, ^9^, ^10^ Some defects in these proteins are serious enough to cause sterility.

Dyneins are one of three major cytoskeletal motors^11^ and have a wide variety of cellular functions.^12^ This minus end‐directed motor is the only known candidate motor for moving cargoes anteriorly in the oocyte, and its function is essential for determination of oocytes.^13^, ^14^, ^15^ Axonemal dyneins comprise the inner and outer arms of the eukaryotic axoneme, and are essential for cilia or flagella wave forms.^16^, ^17^ This function of dyneins contributes to oocyte transport of motile oviduct cilia and sperm movement, which are important for fertilisation. Defects in the function of inner and/or outer dynein arms may result in primary ciliary dyskinesia (PCD).^18^ This disease phenotype results in lung damage, male infertility, and female subfertility.^19^, ^20^

Dynein axonemal intermediate chain 2 (Dnaic2) is a homologue of human DNAI2, which is associated with PCD.^21^ *Dnaic2* is highly expressed in oocytes and sperms. *Dnaic2*‐overexpressed mice show lung damage, male sterility, and female subfertility or infertility. In the *Dnaic2*‐overexpressed mouse, there are structural abnormalities of the outer and inner dynein arms.^22^ Additionally, defects are found in oogenesis and spermatogenesis, indicating a role of *Dnaic2* in gametogenesis.^22^, ^23^ However, no information is available regarding the potential regulation mechanism of *Dnaic2*.

This study aimed to determine the effect of *Dnaic2* in mice with knockdown of *Dnaic2* and to identify the potential regulatory mechanisms involved in *Dnaic2* function. Our study shows the function of *Dnaic2* in the lungs, testis, ovary, and oviduct. We also show an association between Dnaic2 and signal transducer and activator of transcription 3 (Stat3). These findings indicate that *Dnaic2* plays a role in the lungs, testes, and ovaries by regulating Stat3.

## 2 MATERIALS AND METHODS

### 2.1 Mice

CD‐1, C57BL/6, and C57BL/6 × CD‐1 F1 hybrid mice were used in this study. The recipients were 6‐week‐old C57BL/6 × CD‐1 F1 hybrid or CD‐1 female mice that were sterilised by intraperitoneal injection of busulfan (30 mg/kg; resuspended in Dimethyl sulfoxide, DMSO) and cyclophosphamide (120 mg/kg). Controls were obtained by intraperitoneal injection of DMSO. All procedures were approved by the Institutional Animal Care and Use Committee of Shanghai, and were performed in accordance with the National Research Council Guide for Care and Use of Laboratory Animals.

### 2.2 Constructs

A 1872‐bp open reading frame fragment of *Dnaic2* was subcloned into BamH I‐Hind III sites of the pcDNA3.1 mammalian expression vector pcDNA3.1‐*Dnaic2* for in vitro transfection studies. Four *Dnaic2*‐specific shRNA expression vectors, pRS‐U6‐shDnaic2‐SV40‐Puro, were constructed by OriGene. The four sequences and target location of shRNA‐*Dnaic2* are shown in Table S1. As shown in Figure S1, amongst four shRNAs, shRNA2 and shRNA3 were more efficient than the others. ShRNA3 expression vector was selected for further study.

### 2.3 Generation of *Dnaic2*‐knockdown mice

*Dnaic2* KD mice were generated as described by Zhang et al.^22^ Briefly, we first isolated and then cultured female germline stem cells (FGSCs) from ovaries of CD‐1 mice in the short‐term. FGSCs were then cultured for 3‐5 days and were transfected with the pRS‐U6‐*shDnaic2*‐SV40‐Puro vector using TurboFectin 8.0 transfection reagent according to the manufacturer's instructions (OriGene). After 48 h, the FGSCs were transplanted into ovaries of recipients sterilised by chemotherapy. The recipient mice were mated to wild‐type mice. The genotypes were then identified by PCR and Southern blotting. PCR was performed using 1 μg genomic DNA from mouse tails with primer set (sense: 5′‐TCGACCCTGTGGAATGTGT‐3′; anti‐sense: 5′‐GGGCTTGTACTCGGTCAT‐3′) specific for SV40 early promoter. Southern blotting was used to confirm the results of *Dnaic2* KD mice, and was performed according to the method mentioned above.^22^ Finally, western blotting was carried out using rabbit anti‐DNAI2 or anti‐β‐tubulin (see below).

### 2.4 Histological analysis

Testes, ovaries, and lungs from *Dnaic2* KD and wild‐type mice were fixed with 4% paraformaldehyde, dehydrated, and embedded into paraffin‐wax. The tissues were cut into 6 μm thick sections with a microtome and stained with haematoxylin and eosin for microscopic observation.

### 2.5 Cell culture and transfection

NIH 3T3 cells were cultured at 37°C under 5% CO_2_ in a complete medium, Dulbecco's Modified Eagle Medium (Invitrogen, Carlsbad, CA, USA) supplemented with 10% calf serum and 2 mmol L^−1^ L‐glutamine. These cells were transfected with pcDNA3.1‐*Dnaic2* or pcDNA3.1 (negative control) using TurboFectin 8 solution (Invitrogen) according to the recommended protocol.

For detecting the regulatory mechanism of *Dnaic2*, 293T cells were cotransfected with overexpression *Dnaic2* vectors (containing pcDNA3.1‐Dnaic2 and pRS‐Puro vectors), knockdown *Dnaic2* vectors (containing pcDNA3.1‐Dnaic2 and pRS‐Puro‐shRNA‐*Dnaic2* vectors), or control vectors (containing pcDNA3.1 and pRS‐Puro vectors) according to the method mentioned above. The cells were harvested for analysis after transfection for 48‐72 hours.

### 2.6 Incorporation of 5‐bromo‐2′‐deoxyuridine and immunocytochemistry

The 5‐bromo‐2′‐deoxyuridine (BrdU, Sigma, St Louis, MO, USA) was added to the cell culture with 10 μmol L^−1^ final concentration for 2.5 hours and washed 2‐3 times with phosphate buffer saline (PBS) before fixation. To detect BrdU‐incorporating nuclei, DNA was first denatured to expose the antigen by incubating the cells in 2 N HCl at 37°C for 1 hour. The cells were then rinsed three times by 0.1 M borate buffer followed by three times with PBS. The cells were incubated with primary antibody to BrdU (Lab Vision Corporation, Fremont, CA, USA) and corresponding fluorescein isothiocyanate (FITC)‐conjugated secondary antibody. The nucleus of cells was stained by 4′,6‐diamidino‐2‐phenylindole (DAPI) (Sigma).

### 2.7 Growth curve of cells

NIH 3T3 cells were plated onto 24‐well tissue culture dishes. After 1 day of culture, the cells were transfected with pcDNA3.1‐Dnaic2 or pcDNA3.1 (negative control). The number of cells was determined by counting with a haemocytometer on days 0, 1, 2, 3, 4, and 5. Time course was shown in Figure S2.

### 2.8 Immunoprecipitation and western blotting

Protein from testes, ovaries, or cells was extracted using cold lysis buffer containing 50 mmol L^−1^ Tris‐HCl (pH 7.4), 0.1% (v/v) TritonX‐100, 5 mmol L^−1^ EDTA, 10 μg/mL leupeptin, 10 μg/mL aprotinin, and 1 mM phenylmethylsulfonyl fluoride. For immunoprecipitation, 1‐2 mg anti‐DNAI2 (AVIVA, Beijing, China) was incubated with protein A agarose (Sigma) in lysis buffer at 4°C overnight and centrifuged. The precipitate was washed two times with the same buffer and incubated with protein samples, which were first precleared with protein A agarose at 4°C for 30 minutes and centrifuged. The immunoprecipitate was collected by centrifugation and washed with extraction buffer for western blotting. Membranes were exposed to the following antibodies: anti‐ Epidermal growth factor receptor (EGF‐R) (1:100; BD Biosciences Clontech, Palo Alto, CA, USA), anti‐FAK (1:100, BD Biosciences Clontech), anti‐PI_3_K (1:100; BD Biosciences Clontech), anti‐SH_2_B (1:100, BD Biosciences Clontech), anti‐Stat3 (1:100, BD Biosciences Clontech), anti‐Akt (1:100, Santa Cruz Biotechnology, Inc., Santa Cruz, CA, USA), anti‐c‐kit (1:100; Santa Cruz Biotechnology, Inc.), anti‐TGFβ‐RI (1:100; Santa Cruz Biotechnology, Inc.), anti‐p‐JAK‐2 (1:100; Santa Cruz Biotechnology, Inc.), and anti‐p‐Stat3 (1:100; BD Biosciences Clontech). For western blotting, membranes were immunodetected with anti‐DNAIC2 (1:1000), anti‐Stat3 (1:100), anti‐p‐Stat3 (1:100), anti‐PCNA (1:100; BD Biosciences Clontech), or anti‐β‐tubulin (1:1000; Santa Cruz Biotechnology, Inc.).

### 2.9 Molecular modelling analysis

The crystal structure of Stat3 was reported in 2008 (pdb: 3CWG).^24^ Dnaic2 was constructed using the SWISS‐model with the default setting.^25^, ^26^, ^27^ ZDOCK was used to dock Stat3 and Dnaic2.^28^

### 2.10 RT‐PCR

cDNA was synthesised from 1 μg of total RNA using HiScript reverse transcriptase (HiScript II 1st Strand cDNA synthesis kit; Vazyme, China) in a 20 μl volume containing reverse transcription primer, 25 μmmol L^−1^ oligo(dT), 10 μmol L^−1^ random primers. RT‐PCR was carried out with Taq DNA polymerase (TAKARA) in a 10 μl reaction volume on a eppendorf PCR System using the following conditions: 95°C for 5 minutes, followed by 30 cycles of 95°C for 45 seconds, 53°C for 45 seconds and 72°C for 45 seconds, then 72°C for 10 minutes. The primer pairs used for detecting *Bax* were forward, 5′‐GTTTCATCCAGGATCGAGCAG‐3′ and reverse, 5′‐CATCTTCTTCCAGATGGTGA‐3′. Primers used for B‐cell lymphoma‐2 (*Bcl‐2*) were forward, 5′‐CCTGTGGATGACTGAGTACC‐3′ and reverse, 5′‐GAGACAGCCAGGAGAAATCA‐3′. Primers used for *Gapdh* were forward, 5′‐GTCCCGTAGACAAAATGGTGA and reverse, 5′‐TGCATTGCTGACAATCTTGAG.

### 2.11 Statistical analyses

Statistical analyses were performed using GraphPad Prism 5.0 and Statistical Program for Social Science Version 22 (SPSS 22.0). Statistically significant differences were calculated by the Student's *t* test.

## 3 RESULTS

### 3.1 Generation of *Dnaic2*‐knockdown mice

**Figure 1:**
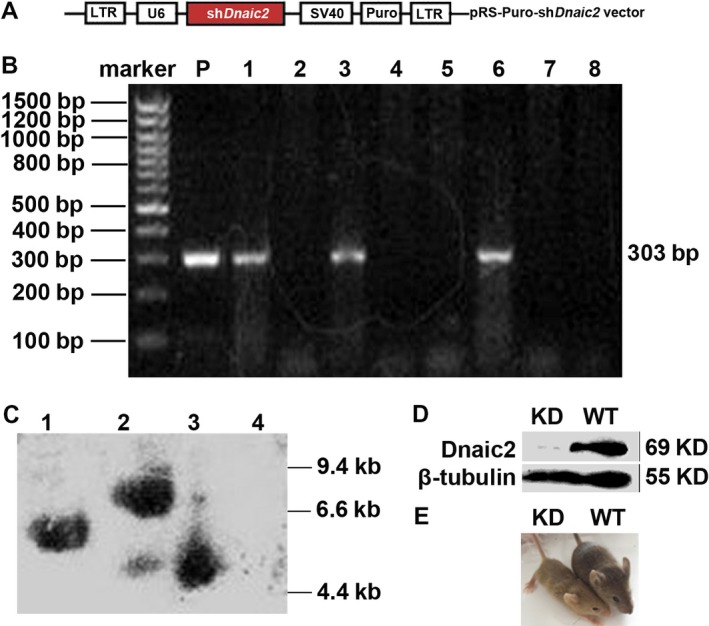
Genotype of *Dnaic2*‐knockdown mice as shown by PCR and Southern blotting. A, Schematic representation of pRS‐Puro‐shRNA‐*Dnaic2* vector constructs that were used for construction of *Dnaic2* KDs. B, PCR analysis for screening *Dnaic2* KDs. Total DNA from the mouse tail was used as a template. Lanes 2, 4, 5, 7, and 8 are samples from wild‐type mice; lanes 1, 3, and 6 are samples from *Dnaic2* KDs. P, pRS‐Puro‐shRNA‐Dnaic2 plasmid. C, Southern blot analysis for screening with a probe, a 303‐bp‐long PCR amplified fragment using primers for SV40 early promoter (see Materials and methods). Lanes 1, 2, and 3 are samples from *Dnaic2* KDs. Lane 4 is a sample from a wild‐type mouse. D**,** Western blot analysis of expression of Dnaic2 in wild‐type or *Dnaic2* KDs. E**,** Images of adult wild‐type and *Dnaic2* KD mice. WT: wild‐type; KD:* Dnaic2* KD mice

To identify the role of *Dnaic2* in PCD and gametogenesis, we generated *Dnaic2‐*knockdown (*Dnaic2‐*KD) mice using the pRS‐U6‐*shDnaic2*‐SV40‐Puro vector (Figure 1A). The offspring of recipient female mice with transplantation were identified as follows. First, PCR was carried out. We detected 128 offspring, of which 36 were *Dnaic2* KD mice (Figure 1B). Southern blotting of three transgenic lines for *Dnaic2* was performed to confirm the results of PCR (Figure 1C). Finally, western blotting was used to show down‐regulation of Dnaic2 protein in the testes, ovaries, and lungs of adult *Dnaic2* KDs (Figure 1D). Bodyweight was lighter and size was smaller in *Dnaic2* KD mice compared to age‐matched wild‐type mice (Figure 1E). We also found that 45% of *Dnaic2* KD mice were infertile.

### 3.2 Phenotype of *Dnaic2*‐knockdown mice

**Figure 2:**
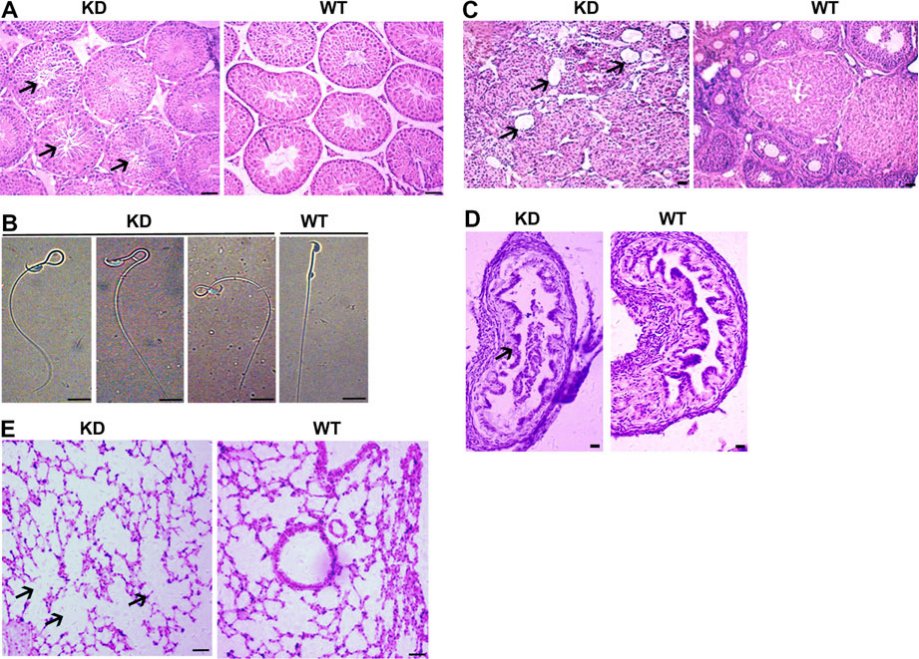
Phenotype of adult *Dnaic2‐*knockdown mice. A, A representive morphology of testis sections from adult wild‐type or *Dnaic2* KD mice after Haematoxylin staining. There are a few sperm in *Dnaic2* KD seminiferous tubules (black arrow). Bar = 50 μm. B, Sperms of *Dnaic2* KD and wild‐type mice. Sperms from adult KD mice were characterised by abnormal morphology, including sperm bent over in the middle part, formation of loops, and the head touching the middle. Bar = 10 μm. C, A representive view of the ovarian sections from adult wild‐type or *Dnaic2* KD mice after Haematoxylin staining. Bar = 50 μm. D, A representive morphology of the oviduct sections from adult wild‐type or *Dnaic2* KD mice after Haematoxylin staining. Bar = 50 μm. E, Haematoxylin‐stained sections of the lungs from adult wild‐type and *Dnaic2* KD mice. WT, wild‐type; KD,* Dnaic2* KD mice. Bar = 50 μm

On the basis of our previous finding that overexpression of *Dnaic2* in mice led to defects of reproduction and lung disease,^22^ we focused our analyses of 2 months old *Dnaic2* KDs on reproductive capacity and histological characteristics of the lungs. Histological analysis showed that fewer elongated spermatids were found in some seminiferous tubules in testes from *Dnaic2* KD mice compared to wild‐type mice (Figure 2A). Additionally, 28.5% of sperm from the epididymis showed an abnormal morphology, including sperm without a head, sperm bent over in the middle part, formation of loops, and the head touching the middle (Figure 2B). Irregular‐shaped follicles were found in the ovaries of *Dnaic2* KD mice compared to wild‐type mice. Moreover, the number of follicles in the ovaries of *Dnaic2* KD mice was less than that in the control groups and no mature follicles were found (Figure 2C). In the fallopian tube lumen of *Dnaic2* KD mice, the mucosal folds were disrupted, ciliated cells had become thinner, some cells had fallen off, and there was a lot of cell adhesions compared to wild‐type mice (Figure 2D). Additionally, histological analysis showed that the pulmonary alveolar structure of the lungs of *Dnaic2* KD mice was damaged, and the alveoli had more serous exudate than in wild‐type mice (Figure 2E).

### 3.3 *Dnaic2* promotes cellular proliferation

**Figure 3:**
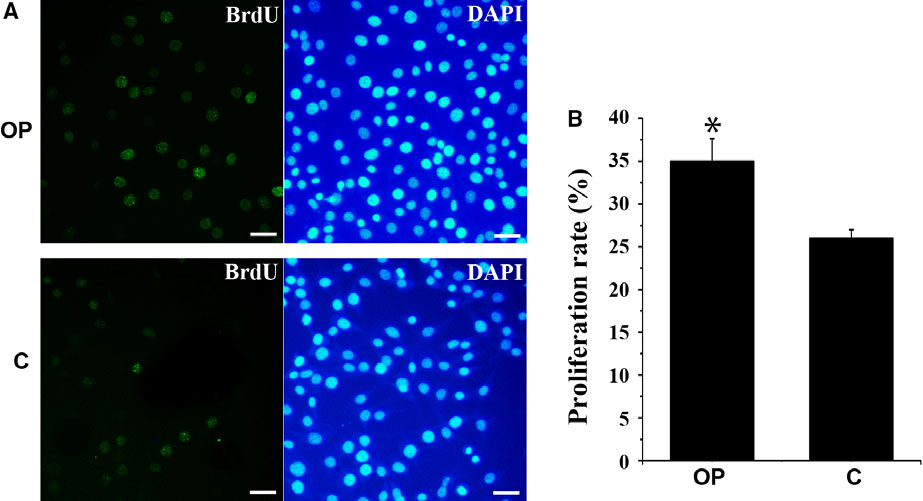
Overexpression of Dnaic2 promotes proliferation of NIH 3T3 cells. A, Cell proliferation assay. The number of cells labelled with BrdU after 2 days with transfection of pcDNA3.1‐Dnaic2 (Dnaic2‐overexpressed vector, OP) was higher than that with transfection of pcDNA3.1 (control, C). Bar = 50 μm. B, Corresponding proliferation rates are represented in bar graphs. Values represent mean ± SEM (n = 3). *P <* *0.05

To determine the effect of *Dnaic2* on the growth of cells in vitro, the proliferation rate of NIH 3T3 cells was detected by BrdU. Two days after transfection with pcDNA3.1‐*Dnaic2*, the proliferation rate of NIH 3T3 cells with overexpression of *Dnaic2* reached approximately 35%, whereas the corresponding control only reached approximately 26% (Figure 3A). Additionally, the growth curve of NIH 3T3 cells with overexpression of *Dnaic2* or control cells was analysed. NIH 3T3 cells that were transfected with pcDNA3.1‐*Dnaic2* grew faster than control cells after transfection (Figure S2), which suggested that *Dnaic2* promoted proliferation of NIH 3T3 cells.

**Figure 4:**
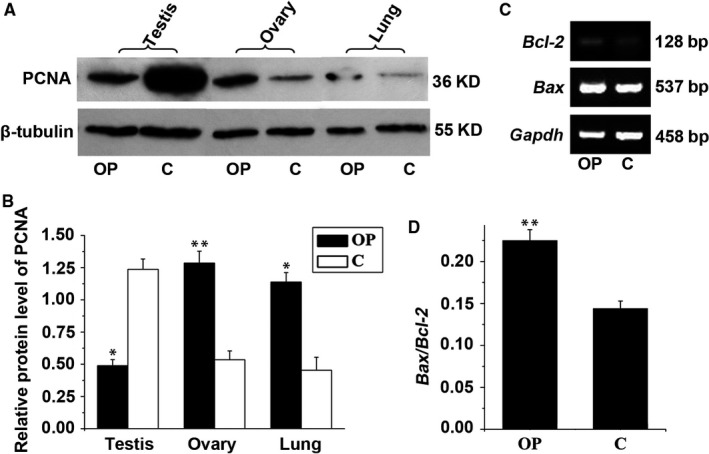
Regulation of cellular fate by Dnaic2 in the lungs, testes, and ovaries of Dnaic2‐overexpressed mice. A, Western blot analysis of expression of PCNA in the testes and ovaries of adult wild‐type or Dnaic2‐overexpressed mice. B, Corresponding bar graphs of panel A. Values represent the relative protein level of PCNA to tubulin (mean ± SEM, n = 3). *P <* *0.05; **P <* *0.01. C, RT‐PCR analysis of expression of *Bcl‐2* and *Bax* in the testes of adult wild‐type or Dnaic2‐overexpressed mice. *Gapdh* was used as a loading control. D, Corresponding bar graphs of panel C. Values represent the mRNA ratio of *Bcl‐2/Gapdh* to *Bax/Gapdh* (mean ± SEM, n = 3). **P <* *0.01. C, wild‐type mice; OP, Dnaic2‐overexpressed mice

To determine if *Dnaic2* activates cellular proliferation in vivo, we analysed expression of PCNA, which is one of the potential proliferation markers in mouse testes, ovaries, and lungs. PCNA protein expression levels in the lungs and ovaries from *Dnaic2*‐overexpressed mice were significantly higher than that in wild‐type mice (Figure 4A, B). This finding suggests that Dnaic2 actives cellular proliferation in mouse lungs and ovaries. Interestingly, a completely opposite result was found in the testes (Figure 4A, B), and this was supported by *Bax/Bcl‐2* results (Figure 4C, D). These findings suggest that *Dnaic2* inhibited cellular proliferation in mouse testis.

### 3.4 *Dnaic2* interacts with Stat3

**Figure 5:**
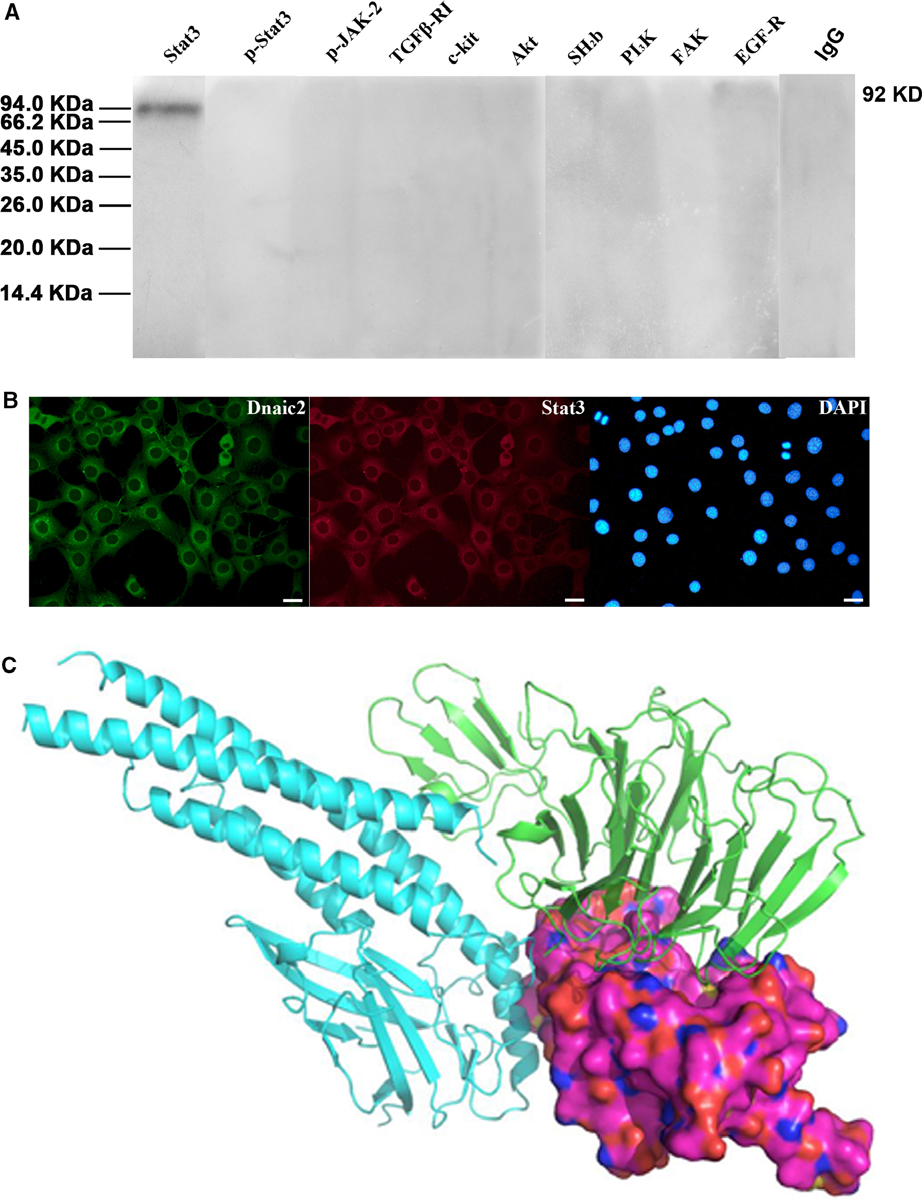
Interaction of Dnaic2 with proteins. A, Immunoprecipitation analysis was performed using lysates of the testes of the adult mouse. Proteins were pulled down by rabbit‐anti‐DNAI2 antibody and then analysed by Western blot analysis. Dnaic2 interacted with Stat3. B, Double‐labelling immunofluorescence cell staining analysis. The co‐expression of Dnaic2 and Stat3 in NIH 3T3 cells. Bar = 25 μm. C, The binding interface between Stat3 and Dnaic2. Green indicates Dnaic2, cyan indicates coil‐coil and DNA‐binding domains of Stat3, stacking surface for linker and SH2 domain of Stat3

To investigate proteins that are potentially associated with *Dnaic2* function, immunoprecipitation analysis was performed. According to the sub‐cellular location and function of *Dnaic2*, 10 proteins were selected to be tested, including the cell surface receptors TGFβ‐RI, EGF‐R, and c‐kit. These receptors are important proteins in regulation of spermatogenesis and follicular development. We examined proteins in the JAK/Stat signaling pathway, which is responsible for signals from the cytoplasm to the nucleus, including JAK‐2, Stat3, and SH_2_B. We also investigated members of the PI3K‐Akt signalling pathway, which participates in transduction of multiple signals, including PI3K, Akt, and FAK. Amongst these proteins, Stat3 was pulled down together with Dnaic2 (Figure 5A), which indicated that Stat3 was a binding partner of Dnaic2. Immunofluorescence double staining analysis showed a consistent expression pattern of Dnaic2 and Stat3 (Figure 5B). Moreover, the docking complex between Stat3 and Dnaic2 is shown in Figure 5C. Dnaic2 was bound with the linker and SH2 domains of Stat3 (Figure 5C).

### 3.5 *Dnaic2* activates Stat3

**Figure 6:**
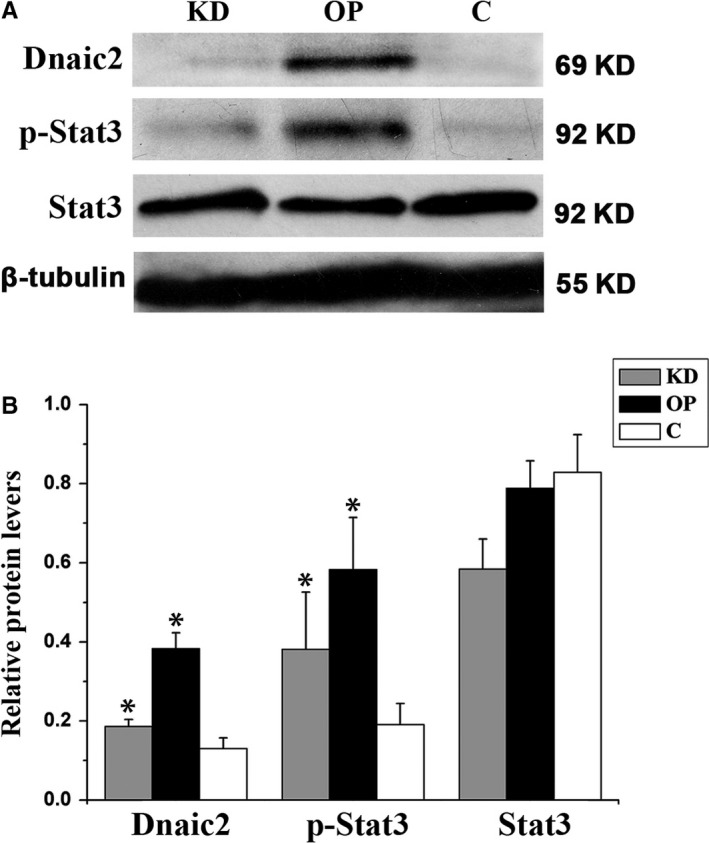
Activation of Stat3 by Dnaic2 in vitro. A, Western blot analysis of expression of Dnaic2, p‐Stat3 and Stat3. Phosphorylation of Stat3 in 293T cells with cotransfection of pcDNA3.1 and pRS‐Puro (C), cotransfection of pcDNA3.1‐Dnaic2 and pRS‐Puro (OP), and cotransfection of pcDNA3.1‐Dnaic2 and pRS‐Puro‐shRNA2 (KD) are also shown. Overexpression of Dnaic2 did not affect Stat3 expression, but enhanced phosphorylation of Stat3. B, Corresponding bar graphs of panel A. Values represent the relative protein levels of protein to β‐tubulin (mean ± SEM, n = 3). *P <* *0.05

**Figure 7:**
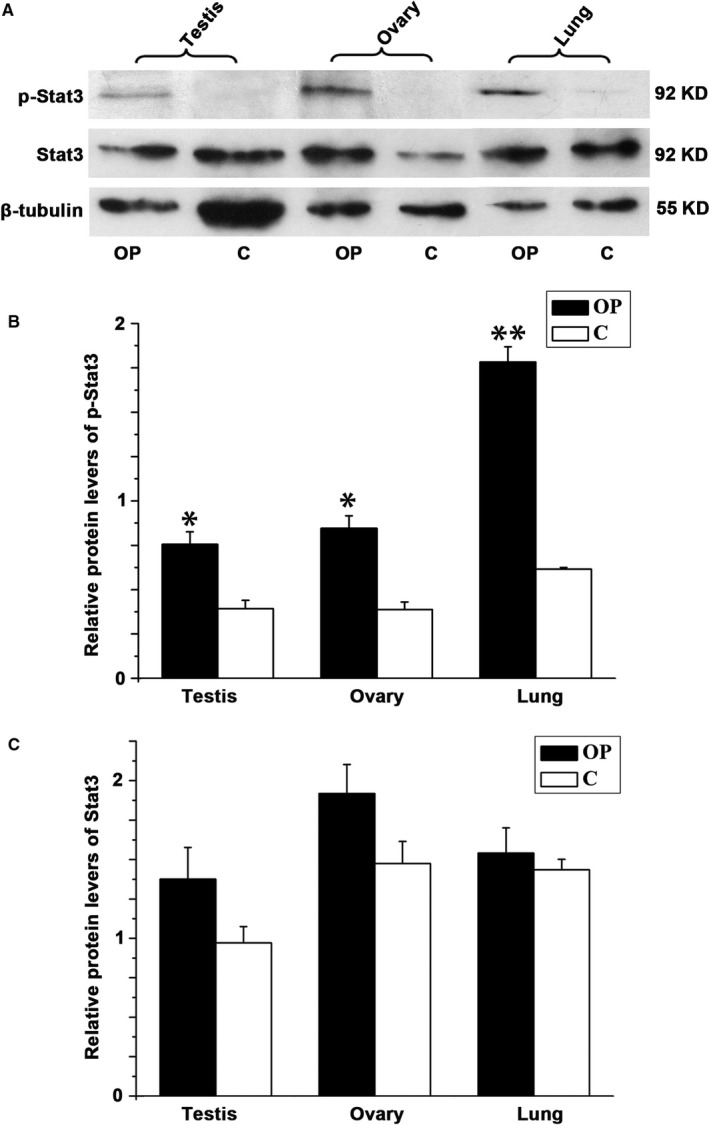
Activation of Stat3 by Dnaic2 in vivo. A, Western blot analysis expression of Stat3 or phosphorylation of Stat3 in the testes and ovaries of adult wild‐type or Dnaic2‐overexpressed mice. Phosphorylation of Stat3 was exacerbated in Dnaic2‐overexpressed mice. (B, C) Corresponding bar graphs of panel A. Values represent the relative levels of protein to β‐tubulin (mean ± SEM, n = 3). *P <* *0.05; **P <* *0.01. C, wild‐type mouse; OP, Dnaic2‐overexpressed mouse

To determine the effect of Dnaic2 on Stat3, we first detected Stat3 expression in the testes, ovaries, lungs, and cells with overexpression of Dnaic2. In 293T cells, Stat3 expression did not change with a change in Dnaic2 expression (Figure 6). However, phosphorylated Stat3 was significantly increased in Dnaic2‐overexpressed cells compared to control cells. Additionally, the inhibition of the expression of Dnaic2 decreased phosphorylation levels of Stat3 (Figure 6). To investigate this situation of Dnaica2 in vivo, we examined expression and phosphorylation of Stat3 in the ovaries, testes, and lungs of *Dnaic2*‐overexpressed mice. Similar results were obtained as in vitro (Figure 7).

## 4 DISCUSSION

In our previous study, we identified *Dnaic2*, and found that it was expressed in mouse ovaries and testes, and played a role in oogenesis and spermatogenesis.^21^, ^22^ *Dnaic2*‐overexpressed mice showed infertility in adults, partly due to some defects of gametogenesis and cilia or flagella in the oviduct or testis. However, not only defects of cilia or flagella occurred, but also disorders of gametogenesis and pathological changes in the oviduct were found in *Dnaic2*‐overexpressed mice.^22^ In the current study, we also observed similar symptoms in *Dnaic2* KD mice, including defects in the testes, ovaries, and oviduct, as well as lung damage. These findings suggested disorder of *Dnaic2* expression, of both overexpression and down‐expression, leading to abnormalities of reproductive development and the lungs. Defects of cilia or flagella may be due to disorder of *Dnaic2* expression, resulting in assembly defects of dynein particles. The outer arm of dynein complexes are disrupted in the *Chlamydomonas oda6* mutant with mutation of the *IC69/IC2* gene^29^ and in respiratory cilia in patients with *Dnaic2* mutations (*IC69/IC2* and *Dnaic2* are orthologous to *Dnaic2* 21, 29, 30). These findings suggest the essential role of *Dnaic2* in assembly of the dynein complex, which controls the motility of cilia or flagella.

The potential regulatory mechanism of *Dnaic2* in gametogenesis or cellular fate in the lungs is unknown. In this study, we detected proteins that are associated with the function of *Dnaic2*. First, we found that *Dnaic2* promoted cellular proliferation in mouse lungs and ovaries, but had an opposite effect on cells in mouse testes, even if it increased cellular growth of NIH 3T3 cells in vitro. These results indicate that the regulatory mechanism of *Dnaic2* in the mouse is complex. Further, our study showed that Dnaic2 was bound with Stat3. In particular, Dnaic2 could induce phosphorylation of Stat3. This finding suggests that *Dnaic2* plays a role in the testes, ovaries, and lungs by regulating Stat3.

Stat3 is a member of the Stat family proteins. Stat3 is activated in diverse signalling systems. In some cases, Stat3 can induce a set of important target genes, whereas in others it may function as a repressor or as a signalling adaptor, but without transcriptional function.^31^, ^32^, ^33^, ^34^ Therefore, the situation of Stat3 can promote proliferation, survival, or apoptosis in certain tissues.^35^, ^36^, ^37^, ^38^, ^39^, ^40^ Because of the multiple functions of Stat3, *Dnaic2* had different effects on the lungs, testes, and ovary in our study. Many previous studies have reported that Stat3 plays essential and multiple roles in the lungs, including acute lung injury and various cancers.^36^ Defects of Stat3 affect cytoprotection of the respiratory epithelium in the mouse during adenoviral infection.^41^ Accumulating evidence has also demonstrated constitutive activation of Stat3 in multiple cancers, and downstream genes of *Stat3* are biomarkers in human lung carcinomas.^36^ In our previous study, cellular proliferation in the lungs from *Dnaic2*‐overexpressed mice was increased.^22^ In the mouse ovaries, Dnaic2 promoted cellular proliferation in our study, which is consistent with the finding that activity of Stat3 is required for mitosis of ovary cells.^42^ Moreover, our previous study showed that the number of mature follicles was decreased in *Dnaic2*‐overexpressed mice and Dnaic2‐overexpressed mice showed female subfertility or even infertility.^22^ A previous study reported that Stat3 participated in regulation of leptin in oocyte maturation in mammals.^43^ Therefore, *Dnaic2* might regulate maturation of oocytes through Stat3, which affects normal follicular development. Dnaic2 inhibited cellular proliferation in the mouse testes by regulating activity of Stat3. In the adult *Dnaic2*‐overexpressed mice, overexpression of *Dnaic2* inhibited cellular proliferation, affected spermatogenesis.^22^ For further study, we will confirm that the opposing effects described between oocytes/lung and testis are STAT3 dependent or not by using condition knockout stat3 mice.

Taken together, our findings and previous studies suggest that Dnaic2 plays an essential role in the lungs, ovaries, and testes through affecting cellular proliferation by regulation of Stat3.

## CONFLICT OF INTEREST

None declared.
