# Supplementary material for: Dynein axonemal intermediate chain 2 plays a role in gametogenesis by activation of Stat3
Source: J Cell Mol Med. 2018 Nov 1;23(1):417–25. doi: 10.1111/jcmm.13945 (PMC6307815; doi:10.1111/jcmm.13945)
Supplement: Supplementary file 1 [file JCMM-23-417-s001.docx]

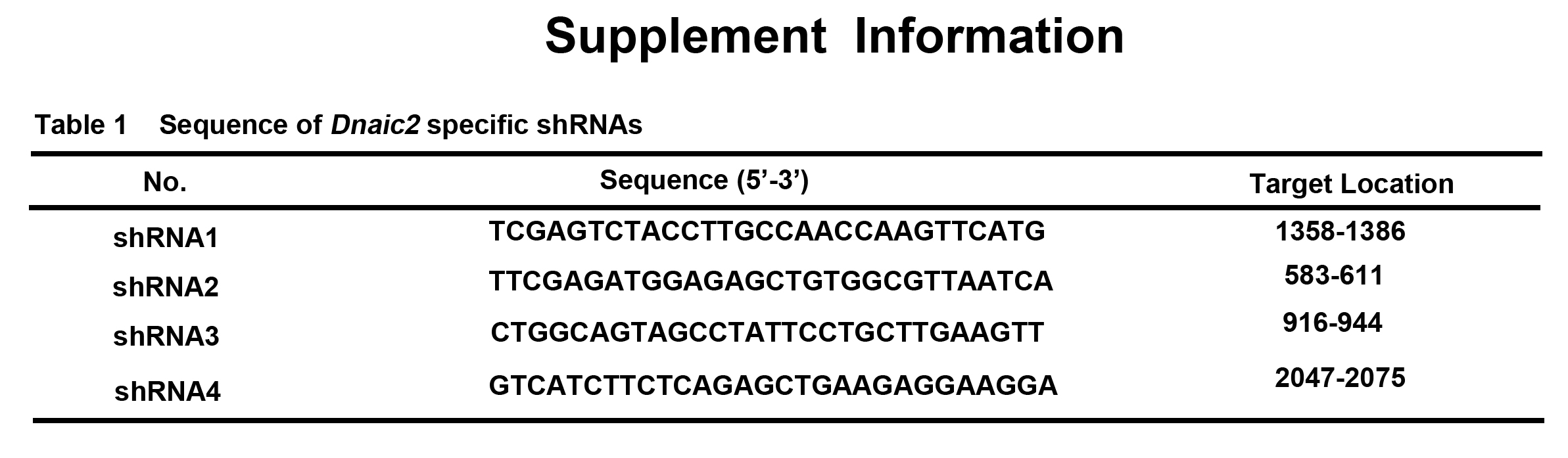


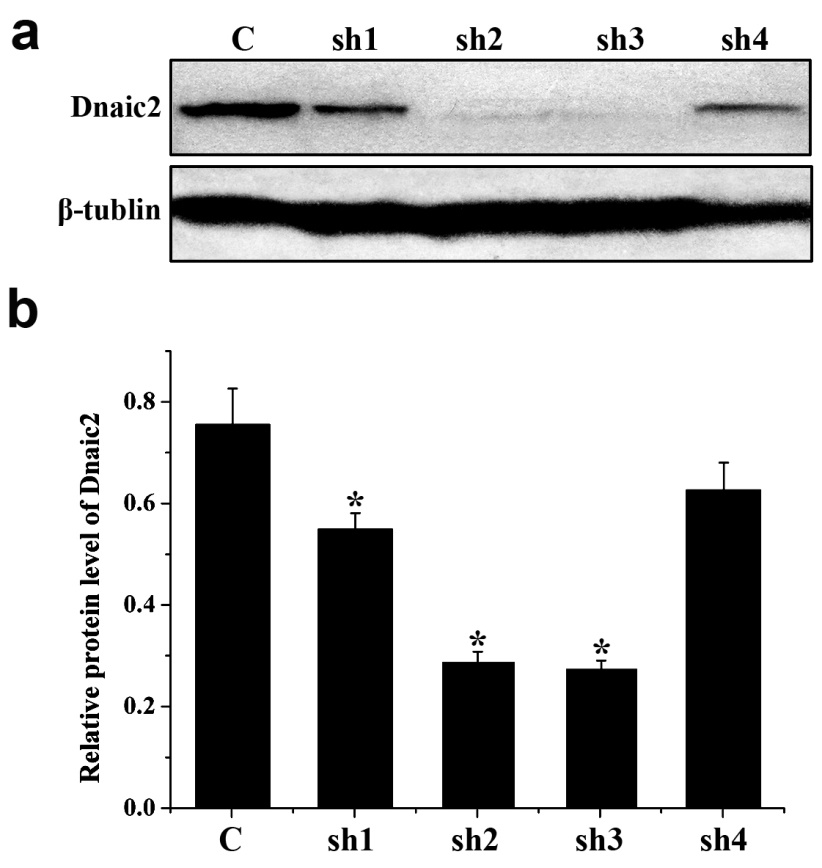


Figure S1

**Figure S1: Dnaic2-specific shRNAs repressed the expression of Dnaic2 protein in 293T cells.** **(a)** Among four shRNAs, shRNA2 and shRNA3 were showed more efficient than the others. Corresponding bar graph is showed in bottom. Values represent the relative protein level of Dnaic2 to β-tublin (mean ± s.e.m., n = 3). Asterisk, *P* < 0.05. C: cell samples co-transfected with pRS-Puro and pcDNA3.1-Dnaic2; sh1: cell samples co-transfected with pRS-Puro-shRNA1 and pcDNA3.1-Dnaic2; sh2: cell samples co-transfected with pRS-Puro-shRNA2 and pcDNA3.1-Dnaic2; sh3: cell samples co-transfected with pRS-Puro-shRNA3 and pcDNA3.1-Dnaic2; sh4: cell samples co-transfected with pRS-Puro-shRNA4 and pcDNA3.1-Dnaic2. **(b)** Corresponding bar graphs of panel A. Values represent the relative protein levels of protein to β-tubulin (mean ± SEM, n = 3). **P* < 0.05.


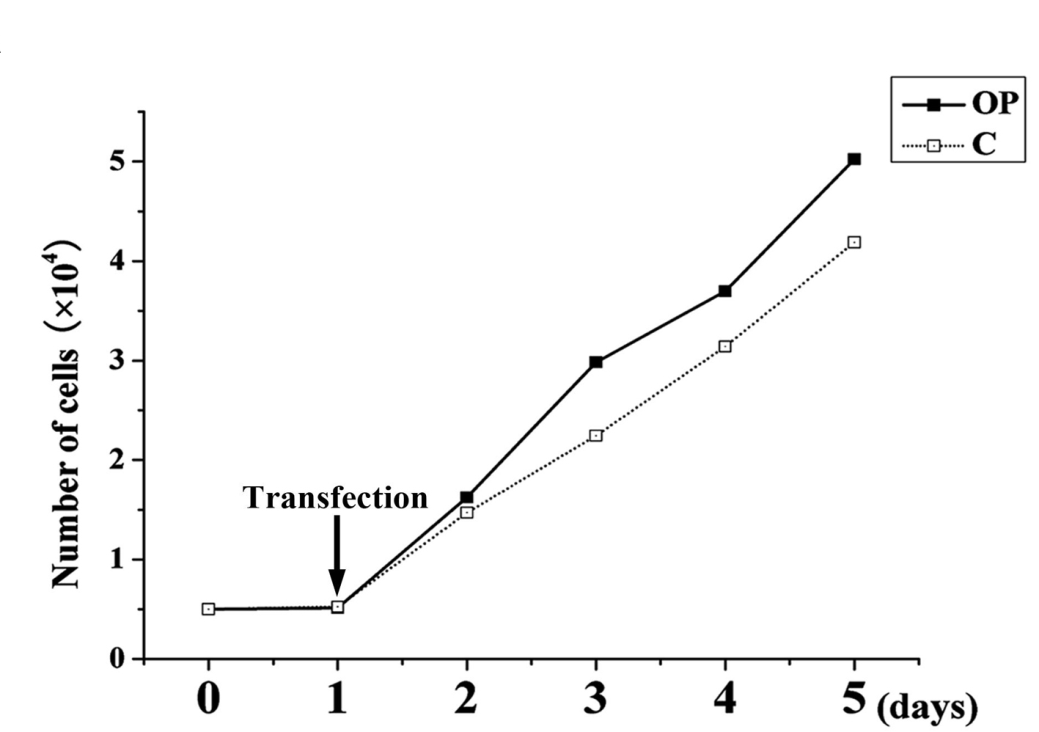


Figure S2

**Figure S2: Overexpression of Dnaic2 up-regulated the proliferation of NIH 3T3 cells.** Growth curve of Dnaic2-overexpressed and control cells was shown. NIH 3T3 cells with Dnaic2-overexpression grew more rapidly than control ones.
